# *Dystrophin* protein and mRNA analyses for the molecular genetic diagnosis of dystrophinopathy: A novel deep intronic *DMD* variant

**DOI:** 10.1016/j.gendis.2025.101557

**Published:** 2025-02-18

**Authors:** Zhiying Xie, Chang Liu, Qingyue Yuan, Zhihao Xie, Meng Yu, Yun Yuan

**Affiliations:** a Department of Neurology, Peking University First Hospital, Beijing 100034, China; b Department of Epidemiology and Health Statistics, School of Public Health, North Sichuan Medical College, Nanchong, Sichuan 637100, China

Becker muscular dystrophy (BMD) and Duchenne muscular dystrophy (DMD) are X-linked recessive muscular dystrophies caused by pathogenic *dystrophin* (*DMD*) variants.^1^ The prevalence of DMD and BMD in Caucasian communities was estimated to be approximately 4∼6 per 100 000 people, which was similar to the prevalence in Asian communities.^2^ Deletions and/or duplications of one or more canonical exons account for ∼80% of disease-causing variants in *DMD*. Most of the remaining ∼20% pathogenic *dystrophin* variants are subexonic small variants, which include small insertions and/or deletions, missense variants, nonsense variants, and canonical splice site variants. Multiplex ligation-dependent probe amplification analysis combined with genomic sequencing of all *DMD* canonical exons and flanking intronic sequences (referred to as the routine DNA-based techniques) can identify most exonic deletions, exonic duplications, and subexonic small variants that occur in *DMD* canonical exons and/or adjacent intronic sequences.^1^ Some rare and atypical pathogenic *dystrophin* variants can escape the detection of routine DNA-based techniques, which mainly consist of complex chromosomal rearrangements and deep intronic splicing-altering variants.^1^ Cryptic exon-activating variants are the most common type among deep intronic splicing-altering variants in *DMD*.^1^ Here, a novel deep intronic cryptic exon-activating variant in the human *dystrophin* gene (NM_004006.2:c.5739 + 404A > G) was identified by skeletal muscle tissue-derived *dystrophin* protein and mRNA analyses, genomic Sanger sequencing, and long-read whole *DMD* gene sequencing.

A 30-year-old male patient, who had progressive muscle weakness of bilateral lower limbs since 25 years of age, was recruited. The level of his serum creatine kinase concentration was significantly increased (5878 IU/L). He now has bilateral tendon contractures, calf hypertrophy, and limb-girdle muscle weakness. However, he has no cardiac muscle involvement in terms of his clinical manifestations, electrocardiogram, and echocardiography examinations. Skeletal muscle biopsy revealed a mild muscular dystrophic pattern (Fig. 1A) and reduced expression of dystrophin-N, –C, and –R (Fig. 1B–D). Western blotting also revealed a reduced expression of full-length dystrophin protein in the recruited patient (6.7% of that found in the healthy control muscle; Fig. 1I). Multiplex ligation-dependent probe amplification analysis of *dystrophin* gene did not detect any deletions and/or duplications of one or more canonical exons in *DMD*. Whole-exome sequencing also did not detect any disease-causing genomic variants in him. Skeletal muscle tissue-derived *dystrophin* mRNA analysis revealed two different transcripts (Fig. 1J). TA cloning successfully distinguished the specific sequences in the two different transcripts; one was the normal splicing of *DMD* exons 40 to 41 (Fig. 1K) and the other was an abnormal 120-bp insertion sequence between *DMD* exons 40 and 41 (Fig. 1L). The abnormal 120-bp insertion sequence was homologous to (100%) a deep intronic region of *DMD* intron 40 (chrX:32360848–32360967) and thus was genetically regarded as a cryptic exon or pseudoexon activation (NM_004006.2:r.[=,5739_5740ins5739 + 284_5739 + 403]). The abnormal cryptic exon transcript, which encoded a frameshift and premature termination codon (NP_003997.1:p.[ = ,Glu1914Metfs∗3]), could be targeted for degradation by nonsense-mediated decay (Fig. 1N).

**Figure 1:**
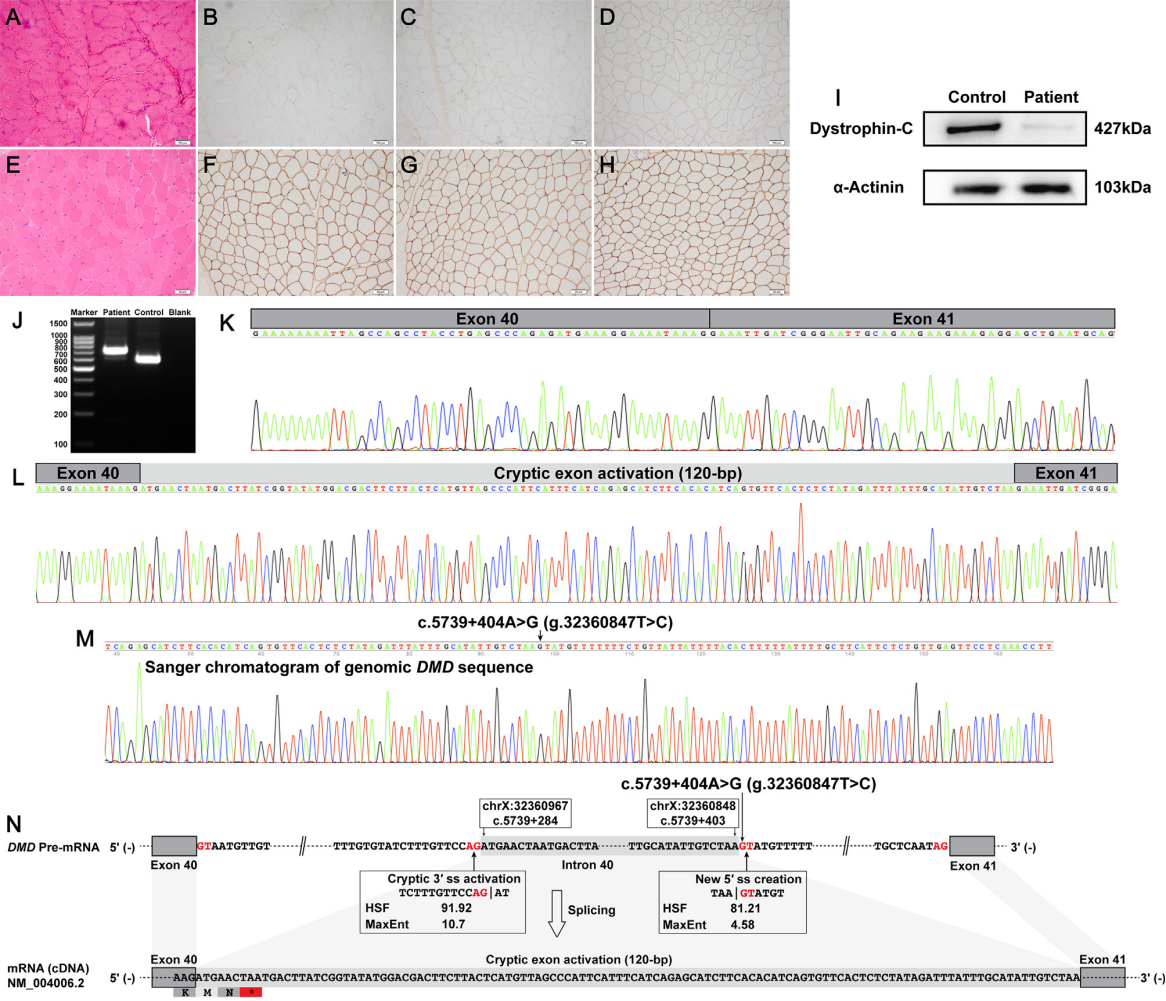
Muscle pathologic findings, *dystrophin* mRNA studies, and genomic Sanger validation of the patient. **(A)** Hematoxylin-eosin staining showed a mild muscular dystrophic pattern. **(B**–**D)** Immunohistochemical staining showed no isolated positive myofibers and traces of dystrophin-N (B), a partial to severe reduction of dystrophin-C (C), and a mild to partial reduction of dystrophin-R (D). **(E**–**H)** Hematoxylin-eosin and immunohistochemical staining showed no histopathologic changes and positive and normal expression of dystrophin protein. **(I)** Western blotting revealed a reduced expression of full-length dystrophin protein. **(J)** Reverse transcription PCR amplification of the abnormal *DMD* splicing transcripts of our recruited patient showed two different bands. **(K, L)** TA cloning distinguished two different transcripts, including the wild-type *DMD* transcript (K) and an insertion sequence between *DMD* exons 40 and 41 (L). **(M)** Genomic Sanger validation of the novel deep intronic variant c.5739 + 404A > G in *DMD*. **(N)** Schematic diagram of the activated cryptic exon caused by c.5739 + 404A > G. (A–D) and (I) are for our recruited patient and (E–H) are for a healthy control. 3′ ss, acceptor splice site; 5′ ss, donor splice site; ∗, premature termination or stop codon; MaxEnt, maximum entropy; HSF, human splicing finder.

Long-read whole *DMD* gene sequencing^1^ of the patient identified a novel and *de novo* single-nucleotide variation around the activated *DMD* cryptic exon (Fig. S1), *i.e.*, the NM_004006.2:c.5739 + 404A > G variant, which was further validated by genomic Sanger sequencing (Fig. 1M). In addition, no pathogenic structural *DMD* variants were identified by the long-read sequencing. The novel c.5739 + 404A > G variant created a new deep intronic 5′ ss (donor splice site) (TAA|GTATGT) and activated a pre-existing cryptic 3′ ss (acceptor splice site; TCTTTGTTCCAG|AT) in intron 40 of the *dystrophin* gene (Fig. 1N). The SpliceAI algorithm predicted the same splicing alterations caused by c.5739 + 404A > G as the Human Splicing Finder and Maximum Entropy algorithms: Donor Gain for the new donor splice site (1-bp) and Acceptor Gain for the pre-existing cryptic acceptor splice site (120-bp) in *DMD* intron 40. The activation of a pre-existing cryptic 3′ ss and the creation of a new deep intronic 5′ ss in *DMD* intron 40 eventually caused the activation of a new *DMD* cryptic exon identified in our recruited patient (Fig. 1N). The details of skeletal muscle biopsy, western blotting, *dystrophin* mRNA analysis, TA cloning, long-read whole *DMD* gene sequencing, and bioinformatic analyses are described in supplementary information.

Pathogenic variants located in introns of protein-coding genes are frequently found to alter pre-mRNA splicing and then cause abnormalities in mRNA and protein. Pathogenic splicing-altering variants account for approximately 9% of all identified pathogenic variants associated with monogenic diseases, including exonic and intronic variants with splicing impact.^3^ Most intronic splicing-altering variants are located in adjacent exon-intron boundaries; hence, they can weaken the strength of essential splicing signals of canonical exons and cause various abnormal pre-mRNA splicing events.^4^ In addition, some intronic splicing-altering variants can also occur in the deep intronic regions of protein-coding genes, and in this condition, they may cause the inclusion of deep intronic sequences into the mature transcripts, which refer to the activation of cryptic exons or pseudoexons. The mechanisms underlying cryptic exon activation mainly include the alterations in splicing regulatory elements and/or essential splicing signals within deep intronic regions.^4^ Deep intronic splicing-altering variants are increasingly reported to cause cryptic exon activation in protein-coding genes associated with monogenic diseases, such as *ABCA4*, *COL4A5*, *DMD*, *CCN6*, *DYSF*, and *COL6A1*.^4^ Among them, the *DMD* gene has a higher occurrence rate of deep intronic splicing-altering variants compared with other monogenic genes, as the *DMD* intronic sequences with an extremely large genomic size provides a bigger target for deep intronic mutations.^4^ To our knowledge, deep intronic splicing-altering variants account for about 3% of all pathogenic *DMD* variants associated with BMD and DMD.^1^ In our study, we found a novel deep intronic splicing-altering variant, the c.5739 + 404A > G variant in *DMD* intron 40, which activated a new *dystrophin* cryptic exon and confirmed the genetic diagnosis of BMD in our recruited patient.

Our study further indicates that muscle biopsy is still an important diagnostic approach for patients with a suspected muscle disease and without a definite genetic diagnosis in the genomic era. Muscle-derived protein and mRNA studies allow the detection and interpretation of nearly all types of pathogenic variants associated with inherited muscle diseases, including atypical missense and synonymous variants, structural variants, non-canonical splice site variants, and deep intronic variants.^1^ Muscle-derived mRNA analysis can also confirm the impact of genomic DNA variants on pre-mRNA splicing to identify abnormalities in splice sites, branch points, or splicing regulatory elements.^4^ In addition, it allows the interpretation of the open reading frame conservation that determines the clinical severity of monogenic diseases. In addition to the main determinant factor (the open reading frame conservation), other factors including epigenetic changes and residual levels of wild-type transcripts should also be considered when interpreting patients' clinical severity. As in our recruited patient, the novel deep intronic *DMD* variant produced an out-of-frame transcript that could theoretically cause a DMD phenotype; however, the residual level of normally spliced *DMD* transcript detected in the patient alleviated his clinical severity and resulted in his BMD phenotype. As skin biopsies are less invasive than muscle biopsies, skin biopsy-derived fibroblasts are a good investigation source for detecting aberrant *DMD* splicing transcripts after converting to myogenic cells,^5^ which might be an alternative tool for mRNA-based genetic diagnosis of BMD and DMD.

Our case study identified a novel deep intronic *DMD* variant via the stepwise application of whole-exome sequencing, dystrophin protein and mRNA analyses, genomic Sanger sequencing, and long-read sequencing, highlighting the significance of intronic *DMD* variants in genetically undiagnosed BMD patients.

## CRediT authorship contribution statement

**Zhiying Xie:** Writing – review & editing, Writing – original draft, Visualization, Validation, Methodology, Investigation, Funding acquisition, Formal analysis, Data curation, Conceptualization. **Chang Liu:** Writing – review & editing, Writing – original draft, Visualization, Validation, Methodology, Investigation, Formal analysis, Data curation. **Qingyue Yuan:** Writing – review & editing, Software, Methodology, Investigation. **Zhihao Xie:** Writing – review & editing, Validation, Software, Methodology, Investigation, Formal analysis, Data curation, Conceptualization. **Meng Yu:** Writing – review & editing, Validation, Supervision, Software, Methodology, Investigation, Data curation, Conceptualization. **Yun Yuan:** Writing – review & editing, Visualization, Validation, Supervision, Investigation, Funding acquisition, Formal analysis, Data curation, Conceptualization.

## Ethics declaration

This study was approved by the Ethics Committee at Peking University First Hospital (approval number: 2023 109–002). The patient provided informed consent for the publication of this case.

## Funding

This work was supported by the 10.13039/501100001809National Natural Science Foundation of China (No. 82201553) and the National High Level Hospital Clinical Research Funding (China) (High Quality Clinical Research Project of 10.13039/100017415Peking University First Hospital, No. 2023HQ10; Scientific Research Fund of 10.13039/100017415Peking University First Hospital).

## Conflict of interests

The authors declared no competing interests.
