# Supplementary material for: Dystrophin protein and mRNA analyses for the molecular genetic diagnosis of dystrophinopathy: A novel deep intronic DMD variant
Source: Genes Dis. 2025 Feb 18;13(1):101557. doi: 10.1016/j.gendis.2025.101557 (PMC12624555; doi:10.1016/j.gendis.2025.101557)
Supplement: Multimedia component 1 [file mmc1.docx]

**Supplementary Information**

# **Supplementary Methods**

## Skeletal muscle biopsy

Skeletal muscle biopsy was obtained from the biceps brachii muscle in the enrolled patient and a healthy control. Routine histological, histochemical, and immunohistochemical staining were performed according to the previously described protocols.^1^ Immunohistochemical staining using a panel of monoclonal antibodies (Novocastra Laboratories, Newcastle) against different domains of dystrophin protein was performed, including dystrophin-N (amino-terminal; DYS3), dystrophin-C (carboxyl-terminal; DYS2), and dystrophin-R (rod-domain; DYS1).

## Western blotting analysis of muscle dystrophin protein

Muscle specimens from the enrolled patient and a healthy control were lysed with SDS buffer containing a cocktail of protease inhibitors (Beyotime). Expression of full-length dystrophin was analyzed by Western blotting using antibody directed against the C-terminal epitope (DYS2; Novocastra Laboratories, Newcastle) of dystrophin protein. α-Actinin (Sigma, A7811) was used as a control for protein loading. The amount of dystrophin protein was normalized against α-Actinin; the resulting ratio was compared with the ratio found in the healthy control muscle tissue, which was normalized to a value of 1.0.

## Muscle-derived *dystrophin* mRNA analysis and TA cloning

Total muscle mRNA was isolated from the remaining muscle biopsy tissue using an RNA extraction kit (Invitrogen, La Jolla, CA, USA) and retrotranscribed to cDNA according to the previous protocols.^1, 2^ Full-length cDNA of the *DMD* gene (GenBank accession number NM_004006.2) was amplified and Sanger sequenced on an 3730XL Sequencer in 22 overlapping fragments using a set of primers (Table S1).^1^ PCR reactions were performed using touchdown PCR. The annealing temperature was dropped by −0.6°C for the first 25 cycles, followed by 20 cycles of optimal annealing temperature for each primer set. Cycling conditions were as follows: 95°C/5 min, followed by 25 cycles (95°C/30 s, 65°C/30 s [−0.6°C per cycle], 72°C/90 s), followed by 20 cycles (95°C/30 s, 50°C/30 s, 72°C/90 s), and followed by final extension at 72 °C for 10 min. The sequences of the 22 overlapping cDNA fragments were aligned to the reference sequence (NM_004006.2) using the CodonCode Aligner 9.0.1.

TA cloning was performed to distinguish the specific sequences in the two different transcripts. The purified PCR products of the two different transcripts were reacted with a T vector assay (pClone007 Cloning Vector Kit, TsingKe, China) and subsequently cloned into competent cells. After a reaction on ice, in a water bath with 42°C, and on ice again, the reaction was incubated in Lysogeny Broth medium. The cells were then spread on the plate and grown overnight at 37°C. Selected colonies were inoculated in Lysogeny Broth/Ampicillin medium and shaken overnight at room temperature. The plasmids were digested with restriction enzymes and subsequently Sanger sequenced using the primers, i.e., M13F (TGTAAAACGACGGCCAGT) and M13R (CAGGAAACAGCTATGACC).^3, 4^

## Long-read whole *DMD* gene sequencing and bioinformatic analyses

Long-read whole *DMD* gene sequencing was conducted using the Pacific Biosciences Sequal I (PacBio Inc., CA, USA) sequencer.^1, 5^ The genomic variants were visualized using the integrative genomics viewer and validated by genomic Sanger sequencing.

The genomic DNA variants, mRNA variants, and protein variants detected in the enrolled patient were described according to the Human Genome Variation Society nomenclature.^6^ Pathogenicity of the detected genomic DNA variants was interpreted and classified according to the American College of Medical Genetics and Genomics (ACMG) guidelines.^7^ The Human BLAT Search tool was used to search genomic sequences (genome build GRCh37/hg19) that were homologous to the detected abnormal splicing transcripts. *In silico* bioinformatic analyses of mutant and wild-type sequences of *DMD* were performed to identify any possible splicing alterations using various algorithms including SpliceAI,^8^ Human Splicing Finder,^9^ and Maximum Entropy Scan.^10^

# **Supplementary Tables**

## Table S1. The list of primers used for the RT-PCR amplification of *DMD* mRNA.

| Amplified fragments | Forward primer sequence (5′ -> 3′) | Reverse primer sequence (5′ -> 3′) |
| --- | --- | --- |
| F1 (exons 1~ 7) | ACTGGAGCAATAAAGTTTGAAGAAC | CTGGCCTATGACTATGGATGAGA |
| F2 (exons 6~10) | AAGATTCTCCTGAGCTGGGTC | CTCCATCAATGAACTGCCAAA |
| F3 (exons 9~13) | GGCTGCTTATGTCACCACCTCT | TTCATCAACTACCACCACCATG |
| F4 (exons 12~17) | AGTACAACAACATAAGGTGCTTCA | AATCCACAGTAATCTGCCTCTTC |
| F5 (exons 17~21) | CACCACTCAGCCATCACTAACA | TAGCCGGTTGACTTCATCCTTA |
| F6 (exons 20~24) | CAGAACAACATCATCGCTTTCTAT | AAAACATCAACTTCAGCCATCC |
| F7 (exons 23~27) | AAATTGAGGGACGCTGGAA | GTGGAGCTTGAGCTATGACACTA |
| F8 (exons 26~30) | CTGTAAGCCTCCAGAAAGAT | CTGCTTGTCAATGAATGTGA |
| F9 (exons 30~34) | GAATCCAGGAGTCCCTCACAT | CAGGCAACTTCAGAATCCAAA |
| F10 (exons 34~37) | ATTGTCCCGTAAGATGCGAAAG | AGCTCTGAGATTTGGGGCTCTA |
| F11 (exons 37~41) | ATACGCCCAAAGGTGGACTC | CCTCAGCTTGCCTACGCACT |
| F12 (exons 40~44) | CAAATTAGCCAGCCTACCTG | GTCAAATCGCCCTTGTCG |
| F13 (exons 43~47) | TATTCATAGCAAGAAGACAGCAGCAT | GCACGGGTCCTCCAGTTTCA |
| F14 (exons 46~50) | GATAACATTGCTAGTATCCCACTT | CTAGGTCAGGCTGCTTTGC |
| F15 (exons 49~53) | AAAACCAGCCACTCAGCC | TGGTGTTCTTGTACTTCATCCC |
| F16 (exons 52~55) | AAAACAAGACCAGCAATCAAG | GAGTCTTCTAGGAGCCTTTCC |
| F17 (exons 55~59) | AAGTTTCTTGCCTGGCTTACA | TCCTCAGCCTGCTTTCGTAG |
| F18 (exons 58~63) | GACAGAGCAGCCTTTGGAAG | TTGTTTGAGTCTCGTGGTTGATA |
| F19 (exons 61~67) | GGACTTTGGTCCAGCATCTCA | GCAACTTCACCCAACTGTCTTG |
| F20 (exons 65~70) | CGGGACGAACAGGGAGGAT | TCTGCACTGGCAGGTAGCC |
| F21 (exons 69~75) | TTGCACTCCGACTACATCAGG | GTGTTGACGCAGTAGCTTGG |
| F22 (exons 75~79) | CTAAAGCAGCAGCACGAACA | CAAATCATCTGCCATGTGGA |

RT-PCR, reverse transcription-PCR. Table S1 has been published in our previous studies.^1, 11^

# **Supplementary Figures**

## Figure S1. The IGV screenshot of long-read whole *DMD* gene sequencing in a normal control and the enrolled patient.


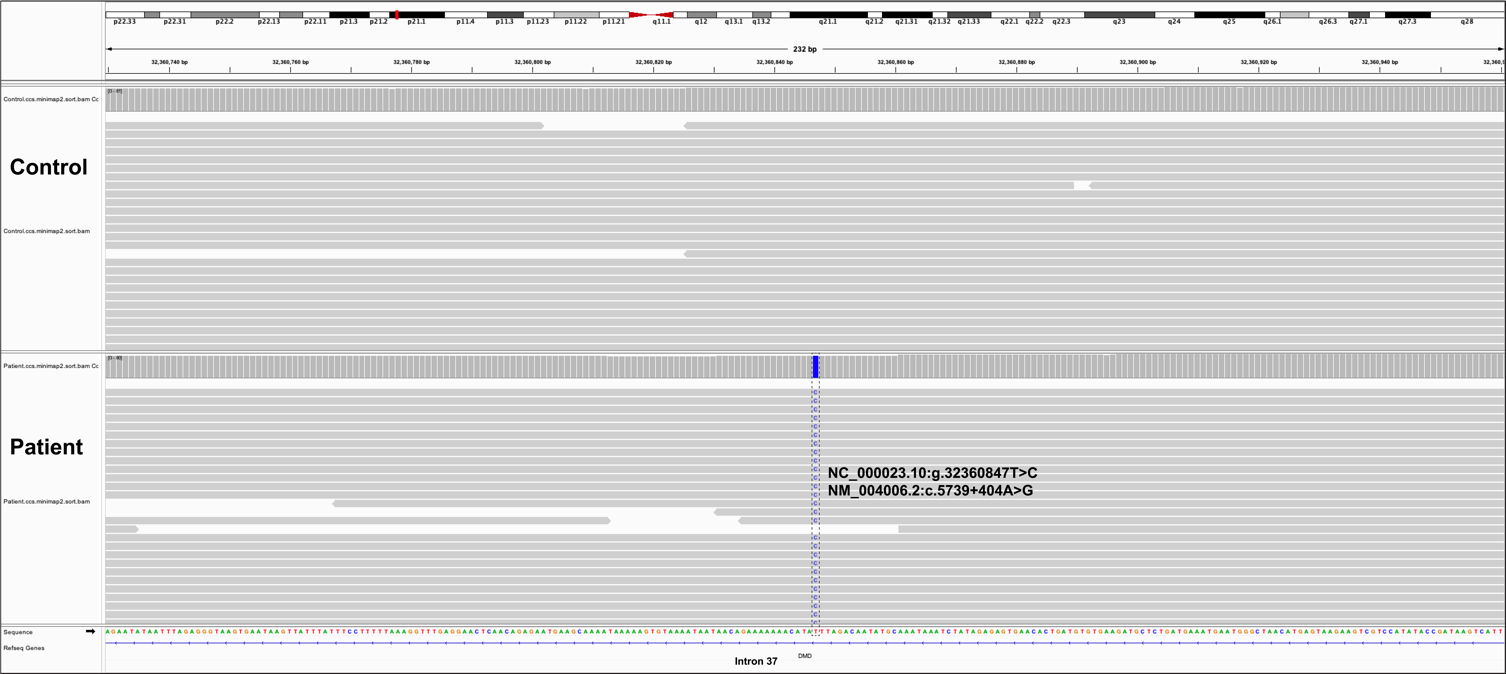


No long reads indicating pathogenic structural variants were found in the enrolled patient and a normal control. In addition to the novel deep-intronic *DMD* variant (NM_004006.2:c.5739+404A>G; NC_000023.10:g.32360847T>C), long-read whole *DMD* gene sequencing did not find other possible small variants responsible for the new *DMD* cryptic exon activation. IGV, integrative genomics viewer.

# **Supplementary References**

1. Xie Z, Sun C, Liu Y, et al. Practical approach to the genetic diagnosis of unsolved dystrophinopathies: a stepwise strategy in the genomic era. J Med Genet 2021;58:743-751.

2. Deng J, Yu J, Li P, et al. Expansion of GGC Repeat in GIPC1 Is Associated with Oculopharyngodistal Myopathy. Am J Hum Genet 2020;106:793-804.

3. Li F, Liu Y, Liu H, Yang J, Zhang F, Feng H. Phenotype and genotype analyses in seven families with dentinogenesis imperfecta or dentin dysplasia. Oral Dis 2017;23:360-366.

4. Xie Z, Sun C, Liu C, et al. Clinical, muscle imaging, and genetic characteristics of dystrophinopathies with deep-intronic DMD variants. J Neurol 2023;270:925-937.

5. Deng J, Gu M, Miao Y, et al. Long-read sequencing identified repeat expansions in the 5'UTR of the NOTCH2NLC gene from Chinese patients with neuronal intranuclear inclusion disease. J Med Genet 2019;56:758-764.

6. den Dunnen JT, Dalgleish R, Maglott DR, et al. HGVS Recommendations for the Description of Sequence Variants: 2016 Update. Hum Mutat 2016;37:564-569.

7. Richards S, Aziz N, Bale S, et al. Standards and guidelines for the interpretation of sequence variants: a joint consensus recommendation of the American College of Medical Genetics and Genomics and the Association for Molecular Pathology. Genet Med 2015;17:405-424.

8. Jaganathan K, Kyriazopoulou Panagiotopoulou S, McRae JF, et al. Predicting Splicing from Primary Sequence with Deep Learning. Cell 2019;176:535-548.e524.

9. Desmet FO, Hamroun D, Lalande M, Collod-Béroud G, Claustres M, Béroud C. Human Splicing Finder: an online bioinformatics tool to predict splicing signals. Nucleic Acids Res 2009;37:e67.

10. Shamsani J, Kazakoff SH, Armean IM, et al. A plugin for the Ensembl Variant Effect Predictor that uses MaxEntScan to predict variant spliceogenicity. Bioinformatics 2019;35:2315-2317.

11. Xie Z, Sun C, Liu C, et al. First Identification of Rare Exonic and Deep Intronic Splice-Altering Variants in Patients With Beta-Sarcoglycanopathy. Front Pediatr 2022;10:900280.
